# Medical and Physical Intelligence

**Published:** 1804-02-01

**Authors:** 


### MEDICAL AND PHYSICAL INTELLIGENCE.

### [ FOREIGN AND DOMESTIC. ]

Dr. Mœrker, of Marienwerder, relates a curious case, in which a solution of emetic tartar was injected into a vein for the purpose of removing a piece of beef that had stuck in the fauces. A man, sixty years of age, taking beef for his supper, which for want of teeth he was not capable of properly chewing, had a piece of it stuck so fast in his throat, that all attempts to bring it up or down were ineffectual. Mr. Knopf, a surgeon, being sent for, found the man in a most deplorable situation; he could hardly breathe, his face was tumid and blue, and he was very near being choaked. After having uselessly endeavoured to draw the foreign body up or to push it down, the surgeon remembered reading in the late Mr. Schmucker's Surgical Cases, of an injection of emetic tartar into the veins having been practised in a similar case with unexpected success; and accordingly, as no time was to be lost, he determined to try the same expedient. To this end he dissolved four grains of emetic tartar in half an ounce of warm water, and having put the solution into a small syringe provided with a long tube, he injected it into the vena mediana of the right arm; having previously stopt the opening of the vein with his finger, and loosened the bandage round the arm, he directed the tube of the syringe upwards; the injection was made slowly, and the liquor had the temperature of the blood. About a minute after this operation the man turned sick, and soon after he began to vomit violently, by which means he brought up a great quantity of pituita and at the same time the piece piece of beef, which was swollen to the size of an egg. The life of Mr. K's patient was saved also by the same means; and the surgeon adds, that after the first vomiting had been over, no other vomition or dangerous symptom ensued.

---

In No. 3 of the Journal de Medicine, Chirurgie, &c. de Montpelier, the following remarkable case of the effects of Galvanism in hydrophobia is recorded. A man, who had been bitten by a mad dog in the middle finger, complained about a month after of pains in the arm and back, but particularly in the finger. On applying the actual cautery the pains disappeared, but some days after returned, and were attended with symptoms of hydrophobia; the patient could not bear the sight of water, and shewed a great inclination to bite. Under these circumstances the patient was exposed to the action of Galvanism; and as he could not suffer the sight of water or any other lucid object, a Galvanic pile of fifty strata of silver, zinc and pasteboard plates was erected in the adjoining apartment; oblong pieces of blotting paper, which were moistened, served as conductors, and on which the patient was placed with his feet naked. At the moment he opened his mouth to bite, one end of a conducting arch was introduced, the other end of which was in connection with the pile. The patient suffered a great deal after each operation, and found himself so fatigued that he was not able to stand. On this account he was laid on the ground, and the application of Galvanism continued; during which time a perspiration came on. It was intended to repeat the operation the following day, but the patient finding himself well, would not undergo it a second time. A few days after some symptoms re-appearing, he willingly suffered himself to be again galvanised; which being done, he never afterwards perceived any symptom threatening a relapse of his former complaint. It is to be remarked that this person, who was naturally very irritable and nervous, felt for above two months after the Galvanic operation, a particular sensation in the body, which extended up to the shoulders.

---

Prof. HUFELAND has found the tinctura cantharidum a most excellent remedy in obstinate cases of hooping cough. It is particularly indicated when the disease is of a more chronic character, attended with atony. It may be given with mucilaginous and bitter remedies, or sometimes with bark, in a dose of from three to eight drops four times a day; but in some cases it is necessary to increase the dose by degrees till a slight burning sensation in making water is perceived. He has likewise found it of great use when united with opium, the effect of which is much increased by this combination.

---

The same gentleman relates the following case of incarcerated hernia, in which he employed the fox-glove with great success. He was called to a young plethoric woman, who had suffered two days days an incarcerated hernia inguinalis, attended with vomition, costiveness, fever, and inflammatory symptoms. She was bled, put into baths; clysters were applied and antispasmodic fomentations, and saline mixtures with oil and opium were prescribed, but all to no purpose; by the opium the fever and heat were considerably increased, and it seemed to be contraindicated by the inflammatory symptoms. He therefore determined to have recourse to the digitalis, which though a strong narcotic and antispasmodic remedy, has the property of acting without heating, and even diminishes the action of the arterial system. He accordingly ordered *one* grain to be taken every three hours with an oily emulsion, and after the patient had continued this medicine for about twenty-four hours, the rupture could be easily replaced by the operation of the taxis, which before had been attempted in vain.

---

It has been observed by Dr. WELPER of Berlin, that the bodies of persons who have been poisoned with arsenic, frequently withstand putrefaction and are changed into the state of mummies. If this fact be confirmed by more experience, it might serve as a sign for discovering previous arsenical poisonings on dead bodies long after death, and it would be worth while to examine the chemical action of that metal on the organic body. At the same time it would lead us to the discovery of an easy and cheap method of preserving dead bodies against putrefaction, or of making mummies.

---

The Lectures of the celebrated Anatomist BOGER upon the Diseases of the Bones, have been reduced to a systematic Treatise by RICHERAND, and will shortly appear in the English language.

---

Dr. KINGLAKE's new work, entitled, A Dissertation on Arthritis or, Gout, exhibiting a new View of the Origin, Nature, Cause, Cure, and Prevention of that afflicting Disease, illustrated and confirmed by a variety of original and communicated Cases, will be published in the course of the ensuing month.

---

Dr. TROTTER's Essay, Medical, Philosophical, and Chemical, on Drunkenness, and its Effects on the Human Body, is in the press, and will be published in a few weeks, being a comment on the Inaugural Dissertation de Ebrietate, &c. Edinb. 1788.

---

Dr. FOTHERGILL, of the Western Dispensary, has in the press a Treatise upon a very singular and painful Affection of the Nerves of the Face, called by certain French Writers and some others, Tic Douloureux.

---

Speedily will be published, Medical Sketches of the Expedition to Egypt by the Army from India, by Mr. M'GRIGOR, the superintending Surgeon of the Indian Army.

